# Driving mosaicism: somatic variants in reference population databases and effect on variant interpretation in rare genetic disease

**DOI:** 10.1186/s40246-021-00371-y

**Published:** 2021-12-14

**Authors:** Vladimir Avramović, Simona Denise Frederiksen, Marjana Brkić, Maja Tarailo-Graovac

**Affiliations:** 1 Departments of Biochemistry, Molecular Biology and Medical Genetics, Cumming School of Medicine, University of Calgary, Calgary, AB T2N 4N1 Canada; 2 Alberta Children’s Hospital Research Institute, University of Calgary, Calgary, AB T2N 4N1 Canada; 3 Department of Neurobiology, Institute for Biological Research, University of Belgrade, 11060 Belgrade, Republic of Serbia; 4 VIB Center for Inflammation Research, Ghent University, 9052 Ghent, Belgium

**Keywords:** Genome sequencing, Blood-derived reference population databases, Rare diseases, Hematopoietic genes, Clonal hematopoiesis of indeterminate potential (CHIP), Cell proliferation

## Abstract

**Background:**

Genetic variation databases provide invaluable information on the presence and frequency of genetic variants in the ‘untargeted’ human population, aggregated with the primary goal to facilitate the interpretation of clinically important variants. The presence of somatic variants in such databases can affect variant assessment in undiagnosed rare disease (RD) patients. Previously, the impact of somatic mosaicism was only considered in relation to two Mendelian disease-associated genes. Here, we expand the analyses to identify additional mosaicism-prone genes in blood-derived reference population databases.

**Results:**

To identify additional mosaicism-prone genes relevant to RDs, we focused on known/previously established ClinVar pathogenic and likely pathogenic single-nucleotide variants, residing in genes associated with early onset, severe autosomal dominant diseases. We asked whether any of these variants are present in a higher-than-expected frequency in the reference population databases and whether there is evidence of somatic origin (i.e., allelic imbalance) rather than germline heterozygosity (~ half of the reads supporting alternative allele). The mosaicism-prone genes identified were further categorized according to the processes they are involved in. Beyond the previously reported *ASXL1* and *DNMT3A*, we identified 7 additional autosomal dominant RD-associated genes with known pathogenic single-nucleotide variants present in the reference population databases and good evidence of allelic imbalance: *BRAF*, *CBL*, *FGFR3, IDH2*, *KRAS, PTPN11* and *SETBP1*. From this group of 9 genes, the majority (*n* = 7) was important for hematopoiesis. In addition, 4 of these genes were involved in cell proliferation. Further assessment of the known 156 hematopoietic genes led to identification of 48 genes (21 not yet associated with RDs) with at least some evidence of mosaicism detectable in reference population databases.

**Conclusions:**

These results stress the importance of considering genes involved in hematopoiesis and cell proliferation when interpreting the presence and frequency of genetic variants in blood-derived reference population databases, both public and private. This is especially important when considering new variants of uncertain significance in known hematopoietic/cell proliferation RD genes and future novel gene–disease associations involving this class of genes.

**Supplementary Information:**

The online version contains supplementary material available at 10.1186/s40246-021-00371-y.

## Introduction

The advent of high-throughput sequencing created a revolution in the discovery and diagnostics of Mendelian diseases [1, 2]. Large amounts of genomic data obtained by exome sequencing (ES) and genome sequencing (GS) are continuously being aggregated to assemble catalogs of ‘normal’ human variation (i.e., a population not affected by severe pediatric conditions or an untargeted population). Examples of such publicly available reference population databases (all blood-derived) include: Exome Aggregation Consortium (ExAC, 60,706 exomes; now merged with the Genome Aggregation Database, gnomAD) [3, 4], gnomAD (v2.1.1 with 125,748 exomes and 15,708 genomes and v3.1 with 76,156 genomes) [4], DiscovEHR (50,726 exomes) [5] and the TOPMed project BRAVO dataset (> 100,000 genomes) [6].

A powerful way to deprioritize potentially non-contributing variants in patients with undiagnosed Mendelian diseases is to assess their presence, frequency and corresponding genotypes in such reference population databases [1, 3, 7]. A variant present in reference population databases with a higher-than-expected frequency for the disease being investigated is considered important support for a benign interpretation [8, 9]. In the past few years, these databases have also been utilized to understand and estimate penetrance in Mendelian disease [1, 7, 10], as well as gene properties (e.g., a gene’s tolerance for loss-of-function (LoF) variants calculated as constraint scores [3, 4]).

While reference population databases offer great power for variant interpretation, the information these databases contain and their limitations need to be well understood to ensure appropriate use. For example, public reference population databases may contain data on some individuals who are not healthy despite the efforts to exclude all individuals with severe pediatric disease (e.g., individual phenotype data may not be fully consented for sharing or carrier individuals may be included due to incomplete penetrance, variable phenotypic expressivity or late disease onset). Moreover, both public and private reference population databases may contain variants of variable quality due to sequencing errors, and despite attempts to apply various filtering strategies for quality control, they may persist and in some instances complicate analyses. Importantly, all of the currently available public reference population databases also suffer from ancestry and diversity biases and hence the efforts to increase the diversity. Some examples of such efforts include the Silent Genomes Indigenous Background Variant Library (IBVL; https://www.bcchr.ca/silent-genomes-project/ibvl), the Greater Middle East Variome project (GME) (http://igm.ucsd.edu/gme/) and the Iranome project (http://www.iranome.ir/). Currently, the reference population databases are based on blood-derived DNA, due to the ease of data collection and quality. Here, we explored the blood-derived reference population data for the presence of somatic variants and their potential impact on diagnostics of rare genetic diseases.

**Fig. 1:**
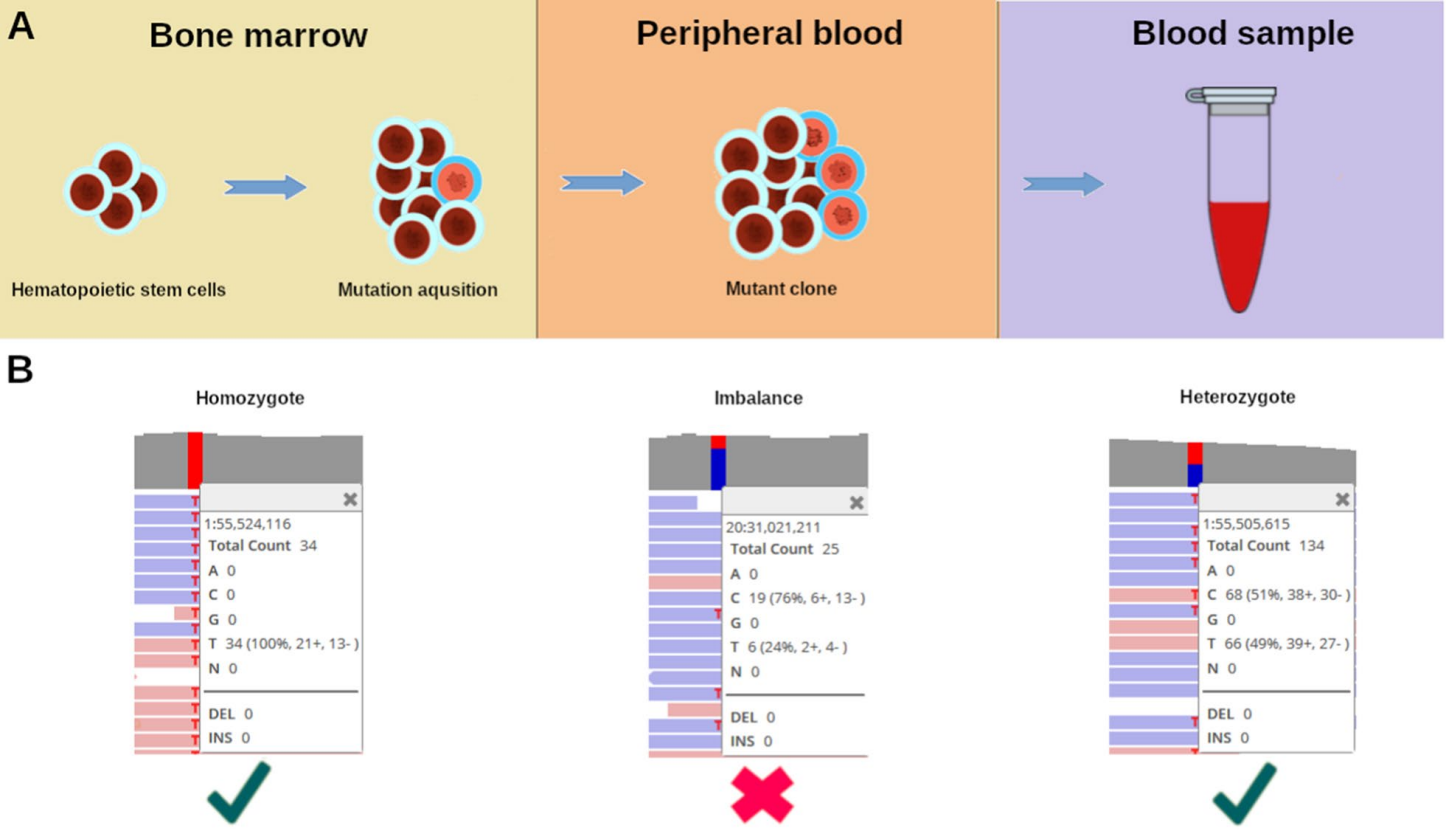
Clonal expansion in Clonal Hematopoiesis of Indeterminate Potential. **A** Somatic driver mutation acquired by a hematopoietic stem cell leads to clonal expansion in the peripheral blood. The mutant clone becomes more abundant in the blood samples, taken as the source of DNA for sequencing. **B** Allelic imbalance as seen in the Integrative Genomic Viewer [37], compared with the examples of true homozygosity and heterozygosity. Recent studies [7, 15] showed that allelic imbalance can be used as an indicator of variant somatic origin

Somatic mutation burden is known to increase with age [11], and somatic variants have also been shown to arise in healthy tissues, especially in those with a rapid turnover such as blood, which has a high mutation load compared with other tissues [11–13]. The function of the genes implicated in mutation load has been associated, not only with healthy tissues, but also with cancer mutagenesis [11]. The variants that confer a growth advantage may allow for ‘clonal expansion’ of a single mutant blood cell, referred to as age-related clonal hematopoiesis (ARCH), or clonal hematopoiesis of indeterminate potential (CHIP) [14] (Fig. 1). This results in the propagation and accumulation of driver variants which can be detected using high-throughput ES and GS data. Sequencing data underlie the reference population databases, and thus, such driver variants may become part of the dataset, potentially complicating variant interpretation (Fig. 1) [7, 15]. Driver variants have previously been described as ‘having such a large impact on fitness that they do not commonly occur in the germline DNA of populations’ [16]. These types of variants (referred to as cancer drivers) have been widely investigated in cancer but may also be the cause of some Mendelian diseases in rare instances when they occur in the germline [17].

Previously, others [15] and we [7] reported unexpected presence of ASXL transcriptional regulator 1 (*ASXL1*) nonsense variants in ExAC. This was unexpected as *ASXL1* haploinsufficiency has been implicated in severe, pediatric, autosomal dominant (AD) disease, Bohring–Opitz syndrome (BOPS [MIM: 605039]). The presence of such *ASXL1* variants in non-BOPS individuals has not been described (i.e., complete penetrance) [7, 9, 15]. Closer inspection of the ExAC dataset suggested that the pathogenic *ASXL1* variants observed in ExAC individuals are of somatic rather than germline origin, likely as a consequence of CHIP [7, 15]. To further understand the effect of mosaicism in blood-derived data and its implications on variant assessment using either public or private reference population data, we searched for additional AD, early onset, severe diseases, like BOPS [MIM: 605039], where well-established pathogenic variants have a higher-than-expected prevalence in a reference population database. Albeit the ExAC database has been merged with the gnomAD database, we decided to use ExAC (v 1.0) for our core analyses. This decision was made to avoid filters applied to gnomAD after the initial publication on *ASXL1* [7, 15] and to get a complete overview of the presence of somatic variants in reference population databases, as some private databases do not use as stringent filtering criteria as those applied in gnomAD.

The main goal of our study was to understand the extent of mosaicism in unfiltered data, so that this knowledge can be generally applied to blood-derived reference population databases (public or private) regardless of filtering strategies and thus improve reliability of variant prioritization workflows.

Here, we identified additional known AD rare disease-associated genes prone to acquiring somatic variants. Our work provides important insights on types of genes that may cause severe pediatric conditions when altered, yet where somatic mosaicism in a reference population may affect variant assessment.

## Results

### Genes associated with autosomal dominant diseases and the presence of ClinVar pathogenic and likely pathogenic SNVs in the ‘untargeted’ population

To learn more about the extent of somatic mosaicism in blood-derived reference population data and its potential effect on variant assessment of early onset, severe AD diseases like BOPS [MIM: 605039], we first assembled a list of 1388 genes implicated in 2010 AD diseases (Additional file 1: Table S1) which we refer to as AD genes. Using this information, ClinVar [18] pathogenic and likely pathogenic SNVs residing in these 1388 AD genes were compiled. Then we asked whether any of these SNVs were present in the reference population database ExAC [7]. We decided to use ExAC (v 1.0) rather than gnomAD as our goal was to understand the extent of mosaicism in unfiltered data. This would make our findings more globally applicable to both public [3, 4, 19] and private reference population databases (https://www.bcchr.ca/silent-genomes-project/ibvl) regardless of the presence and quality of currently used filters.

**Fig. 2:**
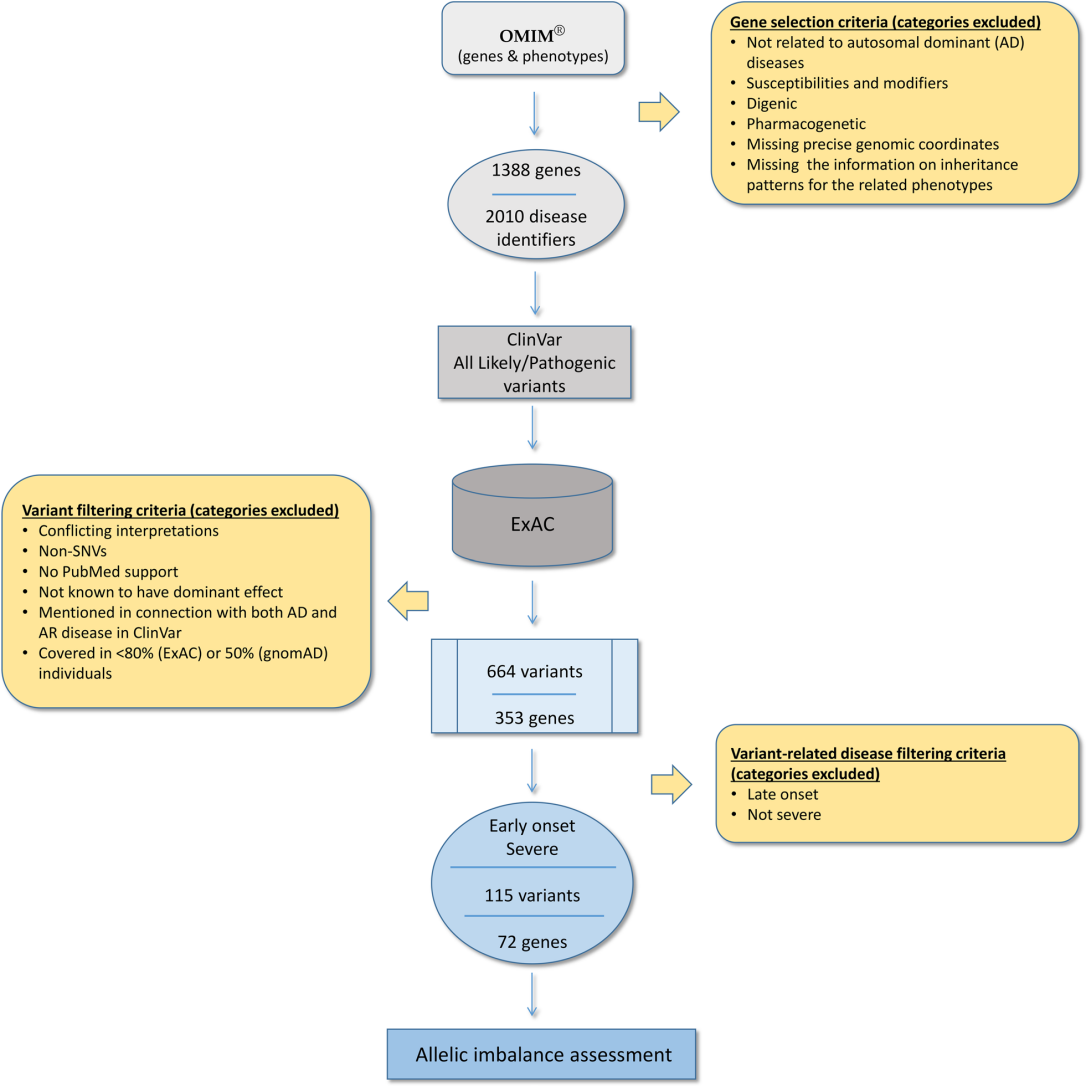
Variant extraction and processing workflow. The extracted variants, present in Exome Aggregation Consortium (ExAC) [3], were assessed for supporting evidence in the current literature (PubMed peer-reviewed articles) and subsequently, for the ratio of reads using the Integrative Genomic Viewer (IGV; used as an indicator of somatic evidence) [37]. For the final nine genes associated with early onset, severe Mendelian diseases and with good evidence of somatic mosaicism, the assessment of allelic imbalance was compared with gnomAD data [4] (Additional file 1: Table S4)

We identified 664 ClinVar pathogenic and likely pathogenic SNVs that are present in individual exomes aggregated in the ExAC database (Additional file 1: Table S2). These SNVs resided in 353 AD genes associated with 390 AD diseases (Fig. 2; Additional file 1: Table S2 and Table 1). Next, we applied the criteria adopted from Chen and associates [10] (Additional file 1: Table S7) to focus on AD diseases characterized as early onset with severe outcomes (with scores of 1 and/or 2) where the presence of pathogenic genotypes in reference population databases could lead to difficulties in variant interpretation and/or delays in diagnosis [7]. The majority of the 390 diseases (~ 80%) was associated with milder or not obvious/life-threatening phenotypes and/or was not early onset (Additional file 1: Table S2) which could explain the presence of individuals with disease-associated genotypes in the ExAC population. However, we found that 115 of the 664 (~ 17%) ClinVar pathogenic and likely pathogenic SNVs present in the ‘general’ population (based on ExAC) resided in 72 genes which were associated with 76 AD early onset conditions, with phenotypes being severe or severe with variable expressivity (Additional file 1: Table S3 and Table 1).

**Table 1:** Summary of analyzed genes, genetic variants and the associated autosomal dominant (AD) diseases

Characteristics	AD conditions		
	All*	Severe and early onset**	Severe and early onset, allelic imbalance***
Number of gene–disease associations	394	77	23
Number of AD diseases	390	76	23
Number of genes	353	72	21
Number of genes per AD disease	1–2	1–2	1
*AD diseases, %*^*β*^			
1–1	1.8	9.1	17.4
1–2	13.5	68.8	60.9
2–1	0.5	2.6	4.3
2–2	3.8	19.5	17.4
Number of variants	664	115	30
Number of variants per AD disease	1–16	1–9	1–3
AD variants, %			
*PubMed articles*			
> 1 peer-reviewed publication	60.4	67.0	80.0
1 peer-reviewed publication	39.6	33.0	20.0
*Variant type*			
Intron	0.3	-	-
Missense	76.7	80.0	80.0
Nonsense	16.4	14.8	16.7
Splice acceptor	2.4	3.5	-
Splice donor	3.3	1.7	3.3
Synonymous	0.8	-	-
Non-coding transcript exon	0.1	-	-
*ClinVar classification*			
Pathogenic	79.7	83.5	90.0
Pathogenic/likely pathogenic	7.1	8.7	10.0
Likely pathogenic	13.2	7.8	-

*AD conditions with ClinVar pathogenic and likely pathogenic ClinVar variants in ExAC (Additional file 1: Table S2)

**Severe and early onset AD diseases with ClinVar pathogenic and likely pathogenic variants in ExAC (Additional file 1: Table S3)

***Severe and early onset AD diseases with ClinVar pathogenic and likely pathogenic variants in ExAC with some evidence of allelic imbalance (at least one allele was found with < 35% of read support; Additional file 1: Table S3). Nine of these genes have evidence of mosaicism (at least two alleles show signs of somatic origin (allelic imbalance))

^β^Age of onset—Severity. Age of onset score 1 means congenital or very early (< 2 years), age of onset score 2 means mostly early (< 18 years) + variable, severity score 1 means severe, significantly reduced mobility or increased mortality in early life, and severity score 2 means severe plus variable expressivity

### Genes associated with early onset, severe autosomal dominant diseases prone to somatic mosaicism and their association with hematopoiesis, proliferation and cancer

Each of the 115 ClinVar pathogenic and likely pathogenic SNVs was assessed to determine whether any of them displayed evidence of somatic mosaicism. Our assessment revealed that at least one SNV in 21 of the 72 genes associated with early onset, severe AD conditions displayed allelic imbalance (Additional file 1: Table S3; Table 1). Nine of those AD genes, including the known genes *ASXL1* and *DNMT3A*, showed the presence of allelic imbalance in two or more alleles, which we define as good evidence of somatic mosaicism (Table 2). The data on age distribution showed that the vast majority of the SNVs that have alleles with imbalanced read ratio come from individuals older than 40 years of age (Additional file 1: Tables S3 and S4). Only 5 of the 30 SNVs that show allelic imbalance in ExAC (Additional file 1: Table S3) have alleles that come from individuals younger than 40 years of age. Further analysis of those nine AD genes with good evidence of somatic mosaicism revealed that most of them (seven genes) play some role in hematopoiesis (as presented in Table 2 based on our cross-analysis with the hematopoietic genes identified by Jaiswal et al. [13]).

**Table 2:** Analyzed genes and variants with their involvement in blood-related functions and cancer

Gene**	AD condition	Variant***	Variant type	Allele count (< 35% of reads)		COSMIC			
				ExAC	gnomAD	AA mutation	Confirmed somatic	No. of samples	Samples being ‘hematopoietic and lymphoid’
*ASXL1* *^▲^	Bohring–Opitz syndrome	NM_015338.5:c.1210C>T^►^	Nonsense	3	3	p.R404*	Yes	10	90.0%
		NM_015338.5:c.2893C>T^►^	Nonsense	1	2	p.R965*	Yes	17	70.6%
		NM_015338.5:c.1117C>T^►^	Nonsense	0	2	–	–	–	–
BRAF*^∆^	Cardio-facio-cutaneous	NM_004333.5:c.1799T>A^►^	Missense	1	2	p.V600E	Yes	29,274	3.0%
	syndrome	NM_004333.5:c.1406G>A	Missense	1	0	p.G469E	Yes	28	0.0%
CBL*	Noonan syndrome-like disorder with or without juvenile myelomonocytic leukemia	NM_005188.3:c.1186T>C	Missense	1	0	p.C396R	Yes	13	100.0%
		NM_005188.3:c.1259G>A^►^	Missense	2	2	p.R420Q	Yes	27	77.8%
		NM_005188.3:c.1111T>C^►^	Missense	1	2	p.Y371H	Yes	30	96.7%
DNMT3A*	Tatton–Brown–Rahman syndrome	NM_022552.5:c.2312G>A^►^	Missense	3	2	p.R771Q	Yes	8	50.0%
		NM_022552.5:c.2644C>T^►^	Missense	4	3	p.R882C	Yes	398	98.5%
		NM_022552.5:c.2536C>T	Nonsense	1	0	p.Q846*	No	1	100.0%
*FGFR3*^▲∆^	LADD syndrome/Thanatophoric dysplasia, type I	NM_000142.5:c.1537G>A	Missense	1	0	–	–	–	–
		NM_000142.5:c.746C>G^►^	Missense	1	0	p.S249C	Yes	1,525	0.0%
IDH2*^∆^	D-2-hydroxyglutaric aciduria 2	NM_001289910.1:c.263G>A	Missense	4	3	–	–	–	–
KRAS*^∆^	Noonan syndrome 3/RAS-associated autoimmune leukoproliferative disorder	NM_004985.4:c.40G>A^►^	Missense	1	0	p.V14I	Yes	34	14.71%
		NM_004985.4:c.35G>A^►^	Missense	1	1	p.G12D	Yes	15,834	1.67%
PTPN11*	Noonan syndrome 1	NM_002834.5:c.1471C>T^►^	Missense	1	NA	–	–	–	–
		NM_002834.5:c.794G>A^►^	Missense	1	0	p.R265Q	Yes	4	100.0%
		NM_002834.5:c.188A>G^►^	Missense	0	1	p.Y63C	No	4	100.0%
*SETBP1*	Schinzel–Giedion midface retraction syndrome	NM_015559.3:c.2608G>A^►^	Missense	2	1	p.G870S	Yes	72	98.61%

A total of 16 variants (80.0% of variants reported here) residing in genes with good evidence of somatic mosaicism were also present in the Catalogue of Somatic Mutations in Cancer (COSMIC; info obtained on February 4, 2021). Across the genes, the variants were found in 1 to 29,274 samples. It is expected that the more frequent a mutation is in cancer samples, the higher the chance is that it is a driver mutation (the mechanism that makes those cells and the variants they carry more abundant in blood). A driver mutation is by definition a genetic change that gives an advantage to the cell. The advantage enables the cell to grow and proliferate better than other cells, which is a hallmark in cancer

AA, amino acid; AD, autosomal dominant; *ASXL1*, ASXL transcriptional regulator 1; *BRAF*, B-Raf proto-oncogene, serine/threonine kinase; *CBL*, Cbl proto-oncogene; COSMIC, Catalogue of Somatic Mutations in Cancer; *DNMT3A*, DNA methyltransferase 3 alpha; *FGFR3*, fibroblast growth factor receptor 3; *IDH2*, isocitrate dehydrogenase (NADP(+)) 2;; LADD, Lacrimo-auriculo-dento-digital; *KRAS*, KRAS proto-oncogene, GTPase; *PTPN11*, protein tyrosine phosphatase non-receptor type 11

^▲^Evidence of somatic mosaicism involving the germline reported by Erickson [39] or Bedoukian et al.[30]

^∆^Genes involved in stem cell and/or cell population proliferation

^►^Variant reported to affect or probably affect function based on Leiden Open Variation Database (LOVD; hg19/GRCh37) version 3.0 [40]

*Genes with evidence of mosaicism that overlap with the list of 156 hematopoietic genes provided by Jaiswal et al. [13] in their Additional file 1: Table S2 (*n* = 7)

**A gene shows **good evidence of mosaicism** when at least two alleles show signs of somatic origin (allelic imbalance)

***Rare disease- and/or cancer-related known pathogenic or likely pathogenic variants (in ClinVar and/or COSMIC)

**Fig. 3:**
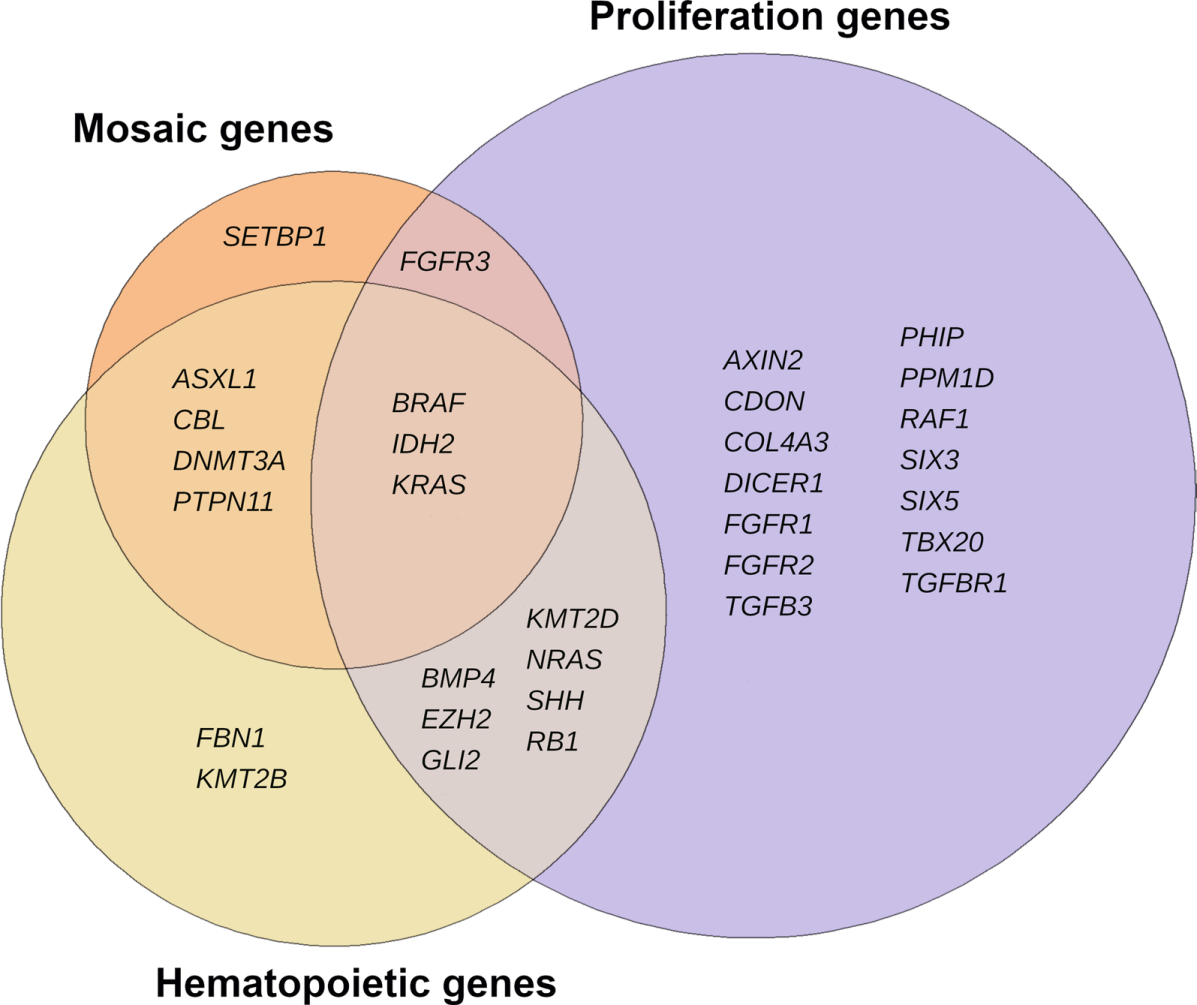
Venn diagram illustrating the classification genes according to the selected feature categories. A total of 16, 9 and 25 genes were considered as hematopoietic, mosaic and proliferative, respectively, based on our categorization [13, 38] and assessment of allelic imbalance [7, 15]. The genes with evidence of mosaicism, *BRAF*, *IDH2* and *KRAS*, belonged to each of the categories (Additional file 1: Table S3). BRAF, B-Raf proto-oncogene, serine/threonine kinase; IDH2, isocitrate dehydrogenase (NADP(+)) 2; KRAS, KRAS proto-oncogene, GTPase;

In addition to the previously known examples of *ASXL1* and *DNMT3A*, the list was expanded to include B-Raf proto-oncogene, serine/threonine kinase (*BRAF*), Cbl proto-oncogene (*CBL*), isocitrate dehydrogenase (NADP(+)) 2 (*IDH2*), KRAS proto-oncogene, GTPase (*KRAS*) and protein tyrosine phosphatase non-receptor type 11 (*PTPN11*), genes known to have a role in hematopoiesis, the formation of cellular components of blood (Fig. 3). The remaining genes with evidence of somatic mosaicism, fibroblast growth factor receptor 3 (*FGFR3*) and set-binding protein 1 (*SETBP1*) were involved in other blood-related functions [14, 20]. Of the AD genes with evidence of somatic mosaicism, *BRAF*, *IDH2* and *KRAS* were, in addition to being hematopoietic genes, involved in regulation of cell population proliferation (GO0042127) based on our gene ontology (GO) categorization (*FGFR3* was also annotated to this GO term; Fig. 3).

Looking further into the driver potential of the SNVs with evidence of allelic imbalance in reference population databases (Table 2), we found that some of these SNVs, known to cause Mendelian disease when inherited via the germline, can be found in the Catalogue of Somatic Mutations in Cancer (COSMIC) database [21], described as associated with malignancies. An example includes the *CBL* variant NM_005188.3:c.1259G>A (p.R420Q), which is known to cause the early onset, severe metabolic disorder Noonan syndrome-like disorder [MIM: 613563]. This variant can be found in COSMIC in relation to cancer; it has been confirmed to be somatic and is predominantly observed in hematopoietic and lymphoid samples (Table 2). We noted similar findings for the other AD genes with evidence of mosaicism, where 16 of the 20 SNVs residing in eight of nine detected mosaicism-prone AD genes were found in COSMIC database (Table 2).

Next, we wanted to compare our findings in nine AD genes with good evidence of somatic mosaicism in ExAC (Additional file 1: Table S3), with gnomAD data (v2.1.1 based on genome build GRCh37/hg19; as presented in Additional file 1: Table S4). The assessment of SNVs residing in the nine AD genes using the gnomAD dataset revealed that some alleles that belonged to *FGFR3*, *KRAS*, *PTPN11* and *SETBP1* SNVs, which had evidence of mosaicism based on ExAC’s exome data, have been removed from the gnomAD dataset by gnomAD filters (Table 2). The following five AD genes showed good evidence of mosaicism in gnomAD, as found using ExAC: *ASXL1*, *BRAF*, *CBL*, *DNMT3A* and *IDH2* (Table 2; with only *DNMT3A* and *IDH2* alleles being flagged for failing of the gnomAD random forest filter). These results support our decision to use the ExAC database as our primary source of information, which allowed us to get a better overview of the mosaicism phenomenon in blood-derived reference populations in general. Furthermore, our findings emphasize the importance for better ways to flag, rather than filter, potentially mosaic variants based on knowledge on the mosaicism-prone genes in reference population databases.

### Somatic mosaicism in other hematopoietic genes

To expand our analysis to other Mendelian diseases (beyond early onset, severe AD diseases) that may be affected by CHIP driver variants, the list of 156 hematopoietic genes compiled by Jaiswal et al. [13] was analyzed. We identified 108 Mendelian diseases with different inheritance patterns which were associated with 77 out of the 156 hematopoietic genes. For the 77 genes, SNVs associated with Mendelian diseases and with evidence of allelic imbalance were selected (Additional file 1: Table S5). By searching the ExAC dataset, we found in total 34 ClinVar pathogenic and likely pathogenic SNVs (nonsense and missense) with read ratio imbalance for at least one allele, residing in these genes. Most of those SNVs (29 of 34 variants) have also been reported as somatic and in relation with different types of cancer in the COSMIC database (Additional file 1: Table S5).

In addition to the already known somatic ClinVar pathogenic and likely pathogenic SNVs in ExAC, we found 125 SNVs of the same variant types (nonsense or missense) which have not yet been associated with a Mendelian disease but have at least one allele with read ratio imbalance, residing in 40 of 156 hematopoietic genes. Some of the hematopoietic genes (besides those nine AD genes we described earlier) have two or more of such SNVs, and 84 of these 125 SNVs have been reported in COSMIC (Additional file 1: Table S5).

The evidence of somatic mosaicism in reference population data and the presence of the majority of these SNVs in the COSMIC database are supportive of a driver potential and thus pathogenicity (Additional file 1: Table S5). This can be explained via examples of the *ASXL1* (for nonsense SNVs) and *DNMT3A* (for missense SNVs) genes. As mentioned earlier, the severe, early onset AD disease BOPS [MIM: 605039] is caused by nonsense *ASXL1* variants. By searching the ExAC dataset, we found two such *ASXL1* SNVs which have been reported in ClinVar, and show allelic imbalance in ExAC (Additional file 1: Tables S3 and S5). At the same time, the ExAC dataset contains additional 20 nonsense *ASXL1* SNVs with some evidence of a somatic origin (allelic imbalance for at least one allele), which are currently not associated with BOPS, but may be in future. The majority of these SNVs (16 of 20 variants) can be found in COSMIC where they were described as somatic and in relation to malignancies (Additional file 1: Table S5). Similarly, some missense *DNMT3A* variants have been reported to cause Tatton–Brown–Rahman syndrome [MIM: 615879], another severe, early onset developmental disease [22]. We found three (two of them missense) *DNMT3A* ClinVar likely pathogenic and pathogenic SNVs, related to Tatton–Brown–Rahman syndrome [MIM: 615879], with read ratio imbalance for at least one allele in ExAC. These SNVs were also reported in COSMIC, in relation to cancer (Additional file 1: Tables S3 and S5). Besides these variants, the ExAC dataset contains 19 *DNMT3A* missense SNVs with some evidence of somatic origin, which are currently not described in relation to Mendelian diseases. Similar to the *ASXL1* example, the majority of these SNVs (14 of 19) have been found in COSMIC where they were reported as confirmed somatic and in relation to different malignancies. In addition to *ASXL1* and *DNMT3A*, we found similar examples in genes such as *CBL*, *IDH2* and *KRAS* (Additional file 1: Table S5)*.* For most of the other genes from the list, ClinVar pathogenic SNVs related to Mendelian disease with evidence of allelic imbalance have not yet been found (Additional file 1: Table S5). Many of those genes, however, harbor somatic SNVs implicated in malignant diseases and are reported in COSMIC (Additional file 1: Table S5).

Overall, when considering both ClinVar pathogenic or likely pathogenic SNVs and the SNVs of the same type, which are still not associated with rare diseases, this sums up to 30.8% of the hematopoietic genes (48 of 156 hematopoietic genes) having at least some evidence of somatic mosaicism (at least one allele with read ratio imbalance in ExAC).

## Discussion

Here, we revealed the presence of somatic ClinVar pathogenic and likely pathogenic SNVs associated with early onset severe AD diseases in the reference population database ExAC for seven additional genes (beyond previously known *ASXL1* and *DNMT3A*). Given the mechanism by which these variants arise in blood tissue, we expect that our findings are applicable to other public and private blood-derived reference population databases. The list of genes prone to acquiring somatic variants will enable variant interpretation computational pipelines to flag variants residing in those genes as ‘potentially somatic’ and thus select them for closer inspection.

A higher-than-expected frequency of variants in an ‘untargeted’ reference population for a given Mendelian disease is generally considered as strong evidence for a benign interpretation when a disease is severe and highly penetrant [9]. Even though the carriers of germline variants associated with such diseases (and their close relatives) usually are recognized and removed from the reference population databases, it is well known that some pathogenic variants are still present [7, 15]. ClinVar pathogenic or likely pathogenic variants of somatic origin (products of CHIP) have been previously detected in these datasets and their potential to affect variant interpretation has been described [15]. However, until now, the only genes associated with Mendelian diseases reported in connection with somatic mosaicism in the context of reference population databases were *ASXL1* and *DNMT3A* [15].

We used the ExAC database with the main goal to avoid the filters applied to the gnomAD database to capture, as comprehensively as possible, genes prone to somatic mosaicism. We found seven additional genes associated with severe, early onset AD rare conditions with good evidence of somatic mosaicism. The majority of these genes has been linked to clonal hematopoiesis (‘expansion of a clonal population of blood cells with one or more somatic mutations’) [23] in the literature (e.g., *ASXL1*, *DNMT3A* and *IDH2*; Additional file 1: Table S4) [13, 23–25]. By profiling the compiled list of 156 hematopoietic genes [13], we were able to identify SNVs with evidence of somatic origin in genes that are associated with the entire spectrum of Mendelian diseases, beyond those with an AD inheritance pattern (Additional file 1: Table S5). This further stresses the importance of considering this mosaicism-prone class of genes when interpreting variants associated with Mendelian diseases regardless of mode of inheritance. Importantly, we also detected somatic SNVs in the reference population, which have not yet been associated with Mendelian conditions. Many of those SNVs have been previously reported as ‘confirmed somatic’ in the COSMIC database in relation to cancer (Additional file 1: Table S5). We propose that for SNVs with unknown significance, the evidence of somatic mosaicism in reference population data, supported by the presence in the COSMIC database, may in fact be considered as a good indicator of their driver potential and thus their potential pathogenic effect in Mendelian diseases, when in germline. Another hallmark of cancer (a prominent form of somatic mosaicism [26]) is sustaining proliferative signaling [27] where increased proliferation has been reported as a consequence of mutations in genes implicated in the hematopoietic system [23]. Seven out of the nine mosaicism-prone AD genes were hematopoietic. Yet, of the remaining genes, *FGFR3* was involved in proliferation (Fig. 3) and *SETBP1* somatic mutations have been connected with myeloid malignancies [28, 29]. This supports our findings providing additional information on a common mechanism by which the somatic variants in these genes arise.

There are several study limitations associated with our work that we would like to highlight to aid in the interpretation of our findings. First, this work was based on the analysis of aggregated data in publicly available reference population databases. Therefore, we did not have access to DNA samples to allow for a direct comparison of sequencing results from different tissues and thus confirm the true genotypes of the individuals. Instead, several important hallmarks of somatic mosaicism were used for characterization of alleles and SNVs. Second, several SNVs with detectable imbalance in ExAC and/or gnomAD were excluded due to low quality (e.g., did not pass quality control filters) which was done to reduce the number of false positives. Nevertheless, some of the potential false positives might in fact be true positives meaning that our study was not able to capture all genes prone to acquiring somatic SNVs. Third, the SNVs without read support available in the IGV were excluded from this study. Those SNVs could potentially have evidence of somatic mosaicism that is not accounted for here. Also, we only focused on the ClinVar pathogenic and likely pathogenic SNVs associated with early onset severe AD conditions in the reference population databases that are due to mosaicism. Other SNVs that do not show signs of mosaicism could be present because (i) they are not pathogenic even though reported as being so, (ii) it was not possible to detect mosaicism using the chosen methodology for those SNVs, (iii) they may not be contributing to AD conditions, (iv) sequencing errors could have occurred that we do not know of and/or (v) occurrence of incomplete penetrance and/or variable expressivity that affects the likelihood of whether a condition develops or not [7]. Finally, as previously described, we used the predefined threshold of < 35% of read support to assess allelic imbalance for the ClinVar pathogenic and likely pathogenic SNVs. However, several studies reported the presence of mosaicism but with 36% of read support [30], and others even report read ratios up to almost 45% for somatic variants, which can be explained by the presence of cancer and aging [15]. Such read ratios were not considered as evidence of somatic mosaicism in our study. Knowing this, it is clear that by applying this threshold we may have lost some valuable data. However, stringent criteria make us more confident that the variants which we focused on in this study are likely of somatic origin. To be even more confident that our selection is not a product of a mere chance (e.g., sequencing errors), we included another criterion, whereby only the existence of at least two alleles with less than 35% of reads is considered as good evidence of somatic mosaicism for a given gene.

Consideration of the identified genes prone to acquiring somatic variants during the interpretation process will be helpful to reduce the risk of errors due to variant misclassification. We showed that the somatic mosaicism is present in large blood-derived publicly available reference population databases, such as ExAC and gnomAD, to a higher extent than it was previously reported [7, 15]. Our preliminary assessments revealed that the same issue applies to another widely used reference population database, TOPMed BRAVO (data not shown) [19]. As we showed by comparing ExAC and gnomAD, the difficulty with the presence of somatic variants in a reference population is not easily solvable by current filtering strategies. Furthermore, our results show that the presence of somatic variants may in fact be a good indication of a driver potential of these variants and thus pathogenicity. Being aware of the potential impact of somatic mosaicism on variant assessment is critical for the successful utilization of these datasets in the variant interpretation process when using either public or private reference population databases such as the IBVL. This private blood-derived reference population database aims to overcome the void of Indigenous population genomics data in the currently available reference population databases, a problem that hinders our ability to efficiently interpret genetic variants from individuals of Indigenous origin. The IBVL, as many other small, private, blood-derived reference population databases, does not have filtering strategies as those employed in gnomAD (which have their own limitations as indicated by our findings). By considering the specific class of genes prone to mosaicism, our work has the potential to help improve variant interpretation practices that rely on blood-derived reference population datasets such as the IBVL.

## Conclusion

We identified 7 additional AD disease-associated genes with recurrent appearance of somatic SNVs in blood-derived reference population databases. In addition to the previously reported *ASXL1* and *DNMT3A*, the list of mosaicism-prone genes was expanded to include *BRAF*, *CBL*, *FGFR3*, *IDH2*, *KRAS*, *PTPN11* and *SETBP1*. All of these genes showed the presence of somatic ClinVar pathogenic or likely pathogenic SNVs in a reference population. Focusing on some of the most widely used blood-derived reference population databases, we showed that the current filtering strategies employed to mitigate the problem of somatic mosaicism were only partially successful. In addition to the variants previously described in ClinVar as pathogenic and likely pathogenic, we revealed other SNVs that show allelic imbalance. Some of these variants are associated with non-AD diseases, while the others are currently not associated with any Mendelian disease, but found in the COSMIC database in relation to cancer and are residing in hematopoietic genes. We expect that at least some of these genes will be associated with Mendelian diseases in the future. Overall, our results underline the importance of considering CHIP and genes involved in hematopoiesis and cell proliferation when interpreting the presence and frequency of genetic variants in both public and private blood-derived reference population databases.

## Methods

### Extraction of genes associated with rare autosomal dominant diseases in OMIM

Genes with at least one phenotype inherited in an AD pattern were identified using gene–disease information stored in the genemap2.txt file (‘May 3, 2019’ OMIM release [22]). The following seven categories were excluded: (i) Non-diseases, indicated by brackets [] in OMIM, (ii) susceptibilities, indicated by braces {} in OMIM, (iii) diseases not inherited in an AD pattern, or digenic diseases, (iv) diseases that are exclusively somatic, (v) pharmacogenetic entries, (vi) modifiers and (vii) loci with no associated HUGO Gene Nomenclature Committee (HGNC) gene, no precise genomic coordinates or without information on type of inheritance for the associated disease. This information formed Additional file 1: Table S1 (Additional file 1: Table S1).

### Variant extraction and filtering using ClinVar and ExAC

Using the AD disease lists (Additional file 1: Table S1), we extracted all ClinVar (VCF v2.0; published on May 3, 2019) [18] pathogenic and likely pathogenic variants that reside in those genes. As previously described [7], only variants with associated publications, namely PMID records (ClinVar var_citations.txt file; October 20, 2020), were considered and assessed for their presence in the ExAC v1.0 (February 27, 2017) [3]. The ClinVar dataset based on the GRCh37/hg19 build was selected to match that of the ExAC database. The variant information was extracted and compared between the ExAC and ClinVar datasets using an in-house Python script. We decided to use single-nucleotide variants (SNVs) as a representative variant type. SNVs account for around 80% of all ClinVar pathogenic or likely pathogenic variants we detected in the ExAC dataset. In addition, compared to some other variant types (e.g., insertions or deletions that include several base pairs), variant read ratio, which was very important part of this assessment, was easier to determine for SNVs. We filtered out any variants that: (i) had conflicting interpretations of pathogenicity in the ClinVar database, (ii) had lack of supporting evidence in the literature, (iii) were not single-nucleotide variants (SNVs), (iv) did not pass ExAC quality filters, or were covered in less than 80% of ExAC individuals, (v) were mentioned in connection with both autosomal dominant and recessive diseases in ClinVar and (vi) were found in the homozygous state in ExAC. This formed a part of Additional file 1: Table S2 (Additional file 1: Table S2).

### Assessment of age of onset and severity of the rare autosomal dominant diseases

For every AD gene–disease association related to the SNVs present in Additional file 1: Table S2, age of onset and severity were graded. This was done according to the criteria adopted from Chen and associates [10] (Additional file 1: Table S7). The AD gene–disease associations were categorized into one of five age of onset categories and one of five severity categories independently by three investigators (M.B., M.T.G. and V.A.). After each investigator completed the evaluation, the results were compared and discrepancies discussed until reaching agreement for all AD gene–disease associations. The evaluation was primarily based on OMIM [22] gene and disease information with support from Malacards [31] and Orphanet [32]. Where sufficient information was lacking, the AD gene–disease association was given a score of 5 (unknown). Only AD gene–disease associations with age of onset and severity scores of 1 and/or 2 were included in the study.

### Variant origin assessment

We evaluated the ClinVar pathogenic and likely pathogenic SNVs associated with early onset (scores 1 and 2) and severe (scores 1 and 2) AD diseases using the Integrative Genomics Viewer (IGV) [33]. Read support count was inspected and the previously published metrics of < 35% alternative allele support [7, 15] was considered as potential evidence of allelic imbalance and thus somatic rather than germline origin (Additional file 1: Table S3). In Additional file 1: Table S3, the ‘Good evidence of mosaicism (ExAC)’ column denotes the presence of allelic imbalance for at least two alleles for a given gene. This criterion was set because allelic imbalance can happen occasionally as a consequence of sequencing errors. However, recurrent appearance of allelic imbalance in a gene is more likely to be caused by the presence of somatic variants. In this sense, we consider the presence of at least one reported allele with imbalanced read ratio for a given gene as ‘some evidence,’ while at least two such alleles per gene were necessary to be considered as ‘good evidence’ of somatic mosaicism. Somatic mutations in blood samples obtained from young individuals are very rare, but rise in frequency with each decade after 40 years of age [13]. The age distribution of individuals with the SNVs of interest was extracted from ExAC and included in Additional file 1: Tables S3 and S4.

### Comparison of ExAC findings with gnomAD data – the effect of the gnomAD filters on the presence and frequency of somatic variants in the selected genes

The ExAC data can now be found as a part of the gnomAD dataset, in addition to other exome and genome data. In contrast to the ExAC database, gnomAD applies more stringent filters that remove some variants with allelic imbalance from the allele count (< 0.2) [4]. To inspect how application of the gnomAD filters affects the presence of somatic SNVs in this reference population dataset, genes with evidence of mosaicism in ExAC (Additional file 1: Table S3) were tested for the presence of ClinVar pathogenic and likely pathogenic SNVs in gnomAD, as previously described for ExAC.

### Gene ontology association

To categorize the genes according to the processes they are involved in, we used selected gene ontology (GO) terms. AD genes associated with early onset severe Mendelian diseases were classified according to their involvement in regulation of hematopoiesis (GO1903706) (Additional file 1: Tables S3 and S4) and involvement in the following cell proliferation processes: Negative regulation of cell population proliferation (GO0008285), positive regulation of cell population proliferation (GO0008284), regulation of cell population proliferation (GO0042127), stem cell proliferation (GO0072089), regulation of stem cell proliferation (GO0072091), positive regulation of stem cell proliferation (GO2000648) and negative regulation of stem cell proliferation (GO2000178). Due to differences in approach, the results from the list of 156 genes published by Jaiswal et al. [13] do not show a complete overlap with the results obtained through our search for regulation of hematopoiesis GO term. However, the results from these sources can be used as complementary to each other. To look up the genes annotated to the aforementioned GO terms, we used the PANTHER [Protein Analysis Through Evolutionary Relationships] Classification System [34–36] and focused specifically on *Homo Sapiens*. The GO database was released on 2020-10-09 and the comparisons were made using the statistical software R 4.0.2.

### Assessment of hematopoietic genes

The total of 156 previously reported hematopoietic genes [13] (Additional file 1: Table S5) were analyzed using ClinVar (pathogenic and likely pathogenic SNVs), ExAC (variant frequency) and IGV (variant read support), as described above (Additional file 1: Table S5). The COSMIC (Catalogue of Somatic Mutations in Cancer) [21] database was used to assess the detected SNVs for reports in human cancers (Additional file 1: Table S5). A detailed description of Additional file 1: Table S5 and other supplementary data (Additional file 1: Tables S1–S5) can be found in Additional file 1: Table S6.

## Supplementary Information

**Additional file 1.** Supplementary tables S1–S7.** Table S1**. Summary of the 1388 genes with the associated autosomal dominant conditions. **Table S2**. Summary of AD genes and the associated conditions with their corresponding ClinVar likely pathogenic and pathogenic SNVs in the ExAC catalog. **Table S3**. Summary of AD genes and the associated early onset, severe conditions with their corresponding ClinVar likely pathogenic and pathogenic SNVs in the ExAC catalog. **Table S4**. gnomAD variant assessment for the genes that show evidence of mosaicism in ExAC. **Table 5**. Mosaicim in hematopoietic genes in ExAC. **Table S6**. Column names and corresponding descriptions for the tables S1-S5. **Table S7**. Phenotype assessment criteria.

## Data Availability

The published article includes all datasets generated or analyzed during this study. The custom scripts used in querying the datasets are available upon request.

## Abbreviations

AD: Autosomal dominant
ARCH: Age-related clonal hematopoiesis
CHIP: Clonal hematopoiesis of indeterminate potential
COSMIC: Catalogue of Somatic Mutations
ES: Exome sequencing
ExAC: Exome Aggregation Database
gnomAD: Genome Aggregation Database
GS: Genome sequencing
GATK: Genome Analysis Toolkit
GO: Gene ontology
IBVL: Indigenous Background Variation Library
IGV: Integrative Genomics Viewer
LoF: Loss-of-function
OMIM: Online Mendelian Inheritance in Men
HGNC: HUGO Gene Nomenclature Committee
SNVs: Single-nucleotide variants
VQSR: Variant Quality Score Recalibration
